# The Unanticipated Challenges Associated With Implementing an Observational Study Protocol in a Large-Scale Physical Activity and Global Positioning System Data Collection

**DOI:** 10.2196/resprot.9537

**Published:** 2018-04-30

**Authors:** Paul McCrorie, David Walker, Anne Ellaway

**Affiliations:** ^1^ MRC/CSO Social and Public Health Sciences Unit University of Glasgow Glasgow United Kingdom

**Keywords:** physical activity, children, data collection, postal survey

## Abstract

**Background:**

Large-scale primary data collections are complex, costly, and time-consuming. Study protocols for trial-based research are now commonplace, with a growing number of similar pieces of work being published on observational research. However, useful additions to the literature base are publications that describe the issues and challenges faced while conducting observational studies. These can provide researchers with insightful knowledge that can inform funding proposals or project development work.

**Objectives:**

In this study, we identify and reflectively discuss the unforeseen or often unpublished issues associated with organizing and implementing a large-scale objectively measured physical activity and global positioning system (GPS) data collection.

**Methods:**

The SPACES (Studying Physical Activity in Children’s Environments across Scotland) study was designed to collect objectively measured physical activity and GPS data from 10- to 11-year-old children across Scotland, using a postal delivery method. The 3 main phases of the project (recruitment, delivery of project materials, and data collection and processing) are described within a 2-stage framework: (1) intended design and (2) implementation of the intended design.

**Results:**

Unanticipated challenges arose, which influenced the data collection process; these encompass four main impact categories: (1) cost, budget, and funding; (2) project timeline; (3) participation and engagement; and (4) data challenges. The main unforeseen issues that impacted our timeline included the informed consent process for children under the age of 18 years; the use of, and coordination with, the postal service to deliver study information and equipment; and the variability associated with when participants began data collection and the time taken to send devices and consent forms back (1-12 months). Unanticipated budgetary issues included the identification of some study materials (AC power adapter) not fitting through letterboxes, as well as the employment of fieldworkers to increase recruitment and the return of consent forms. Finally, we encountered data issues when processing physical activity and GPS data that had been initiated across daylight saving time.

**Conclusions:**

We present learning points and recommendations that may benefit future studies of similar methodology in their early stages of development.

## Introduction

Although there has been a strong movement to publish study protocols within the sociobehavioral sciences before or during data collection [1,2], particularly for trials [3] and more recently larger scale observational work [4], there is very little published material that attempts to describe how the specific protocol was implemented, the hitherto unidentified challenges encountered, and lessons learned. This reflective process is commonplace in the stages of trial/intervention work where a high degree of testing is usually built in during the design, using the lessons learned to refine the intervention [5]. It is also required when conducting rigorous evaluation of a new treatment or public health intervention, including the assessment of outcomes and processes (eg, implementation, mechanisms, and context) [6,7]. It is important for those who are involved in observational research, particularly large-scale national survey work, to build more of these reflective processes into their design. With more of such work being published, researchers can gain insightful knowledge that can be translated to other contexts, which in turn may prevent similar mistakes from being made, thereby minimizing time-consuming unexpected work.

The main aim of this study is to share the experiences gained when conducting a large scale, nationally representative study collecting physical activity and global positioning system (GPS) data from 10- to 11-year-old children across a whole country (Scotland). To do so, this study describes the original methods and processes of the project with particular reference to the recruitment, delivery of project materials, and data collection phases. We then present the issues and challenges experienced when trying to implement our intended design and conclude with recommendations for future research.

## Methods

### Intended Study Design

The study we describe here, Studying Physical Activity in Children’s Environments across Scotland (SPACES), aimed to investigate the ways in which the built environment influences children’s physical activity. The project employed an observational, cross-sectional design that sampled from the Growing Up in Scotland study (GUS; [8]). SPACES data was collected between May 2015 and May 2016 by the Medical Research Council (MRC) and Chief Scientist Office (CSO) funded Social and Public Health Sciences Unit (SPHSU), University of Glasgow. Ethical approval was gained from the College of Social Sciences, University of Glasgow (CSS:400140067).

### Participant Selection—SPACES (Studying Physical Activity in Children’s Environments Across Scotland) Sample

GUS is a nationally representative ongoing longitudinal cohort study that began in 2005 with the aim of tracking the lives of Scottish children. The Birth Cohort 1 (BC1; n=5217) was the first of 2 GUS birth cohorts to have been followed up from age 10 months (sweep 1) until 10 years old (sweep 8 was conducted throughout 2014 and finished in February 2015). The BC1 cohort is split across 2 academic years. The SPACES project included children who started Primary 6 (age approximately 10 years old) in August 2014 (approximately three-fourths of the full GUS BC1, n=2402). As part of the GUS age 10 and 11 interview sweep, parent or carers (n=2402) were provided with brief information about SPACES and asked if their contact details could be passed on to SPACES staff.

### Recruitment

Due to the considerable budgetary and logistical constraints associated with a country-wide study, the primary method of communication was by post. Although we did use other forms of communication (eg, email, text messages [short message service, SMS], and phone) throughout the study, we wanted to maintain a consistent method that minimized burden on the participant (ie, having to use personal printers to print consent forms). Initial recruitment was based primarily on those members (parents and children) of the GUS cohort who consented to being contacted by SPHSU. The details of the GUS participants willing to be contacted by SPACES were sent by ScotCen Social Research—the GUS study management body—to a dedicated member of the project team at SPHSU (database manager). These details included the following: parent and child's full names, postal address, and telephone number.

**Figure 1:**
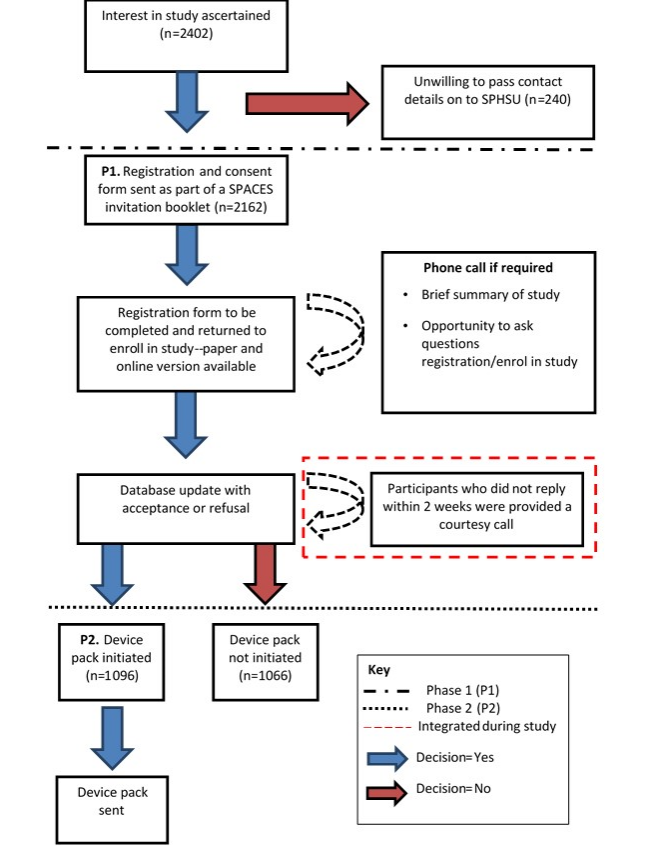
Flow diagram of participant recruitment and registration. SPACES: Studying Physical Activity in Children’s Environments across Scotland; SPHSU: Social and Public Health Sciences Unit.

For those willing to be contacted, we sent invitation packs, by post (P1 in Figure 1), in waves (approximately 200-300 per wave) starting in May 2015 and finishing in November 2015 (excluding the months of July and August for school holidays), containing a letter for the parent and one for the child participant; an information booklet (Multimedia Appendix 1) including a consent form; and a registration document (Multimedia Appendix 2). The registration document was provided in paper format, but the participants were given an option, stated within the information booklet, to complete this via an SPHSU maintained secure webpage on the Internet. The form provided space to request a phone call for further information, or if this was not required, space was given to register for the next stage of the process (P2 Device pack). As part of this document we also asked for an approximate measurement of the participant’s waist size (for the elasticated belt that would hold study devices), and whether a mobile contact number could be requested for SMS reminders. Finally, we asked the parents/participants to propose a start date for the measurement period. To progress to the P2 phase, parents/participants were required to complete and return the registration document.

### Delivery of Project Materials—Device Pack Contents

As part of this phase of the project (P2, Figure 1), we sent participants all the necessary equipment and survey materials to complete the study protocol. The following sections provide brief information on the content of these packs.

### Objectively Measured Physical Activity—The ActiGraph GT3X+

The ActiGraph GT3X+ (ActiGraph, Pensacola, FL, USA) is a validated activity monitor [9,10] that measures acceleration in three orthogonal planes. The device is small (4.6×3.3×1.5 cm), lightweight (19 g), and unobtrusive; it is one of the most widely used monitors in the physical activity field and has been calibrated to accurately capture physical activity of varying intensity (eg, light and moderate) and also sedentary time [11]. Participants were asked to wear the ActiGraph on an elasticated waist belt, during waking hours, for 8 consecutive days. Devices were set to sample acceleration data 100 times per second (100 Hz). This device and the belt were included in the device pack.

### Spatial Measurement—The Qstarz BT-Q1000XT Travel Recorder

The Qstarz BT-Q1000XT travel recorder is a validated GPS device [12], measuring 7.2×4.6×2.0 cm, that records the location of physical activity. Recognized for its acceptable static and dynamic accuracy [12], the device has sufficient battery life, which suited the requirements of our study. The participants were asked to wear the device concurrently with the ActiGraph monitor (held in a “pouch” attached to the same belt), during waking hours, for 8 consecutive days. The devices were set to record location (x/y coordinates) and supporting information (eg, number of visible satellites, elevation) at 10-second intervals. The GPS devices were required to be charged overnight throughout the study period and were set to stop recording when storage capacity had been reached (as opposed to rewriting over that which had already been stored). To assist, each device pack contained a charging cable (universal serial bus, USB) and UK AC adapter (with USB connection). Participants received the devices switched off and were instructed to turn them on when starting the measurement period.

### Self-Reported Physical Activity—Physical Activity Questionnaire-Children (PAQ-C)

Participants were asked to complete the Physical Activity Questionnaire for Children (PAQ-C) following their activity monitoring period (Multimedia Appendix 3). The PAQ-C is a 7-day recall questionnaire that measures habitual levels of physical activity. Validated in children of similar ages [13], the questionnaire was chosen to reflect the cognitive abilities of the age group. It takes approximately 20 min to complete and was available to complete online, or by paper and pen.

### Wear Time Log and Travel Diary

Participants were also provided a log booklet and asked to fill in the times where the accelerometer and GPS were not worn. This allowed us to identify periods in the accelerometer data files where the software would otherwise assume that the participant had been sedentary. The participants were also asked to complete the travel diary by recording the journey mode and associated time when traveling to and from school (Multimedia Appendix 4).

### Instruction Guide

Each participant was sent a short instruction guide for wearing both activity monitor and GPS device (Multimedia Appendix 5). The activity monitor was instructed to be worn with the monitor placed above the right hip and the GPS device above the left hip. The guide included answers to frequently asked questions and specific instructions to charge the GPS device overnight. Our pilot indicated that the GPS devices would have enough storage capacity to record for the 8-day duration of the study and as such participants were initially asked to switch devices on before their first day of data collection and leave them in this position for the remainder of the study. The guide documented the process required to switch the GPS devices to record.

### Data Collection and Processing

A dedicated room with secure access was employed throughout the project, equipped with necessary equipment, including desktop computers. Each computer was installed with the necessary software (ActiLife v.6.11.9 and QTravel V1.48) to initialize and download data from the devices. Fieldworkers were involved in the project at specific times to assist with particular tasks, for example, the period where participants were required to be contacted by telephone at the invite stage of the process.

Our data collection timeline was initially based on a 4-week issue/return cycle. This was put in place to allow us to plan the time required to collect the data for a full participating cohort, based on a working stock number of 400 devices (ie, 400 accelerometers and 400 GPS devices) and contingency of 25 devices for any technical issues/device losses that may have arisen and necessitated replacement during data collection. Devices plus accompanying instructions, time log sheet, and prepaid return envelope were sent out by Royal Mail 1st class post. Participants set their own preferred start date as part of the completed registration document, and our processes were matched to meet these dates. To act as a reminder to wear the devices and increase compliance with the protocol, SMSes were sent midway through the projected device wear period (based on confirmed start date by participant) to parents who provided their details during registration.

## Results

### Issues and Challenges Experienced—Ethics and Informed Consent

In light of the United Nations Convention on the Rights of the Child [14], the research team wanted to ensure that the children of the study (all younger than 16 years) were actively involved in the full consent process: both participants and parents were required to read study documentation, initial consent substatements (eg, consent to share data, or access previously collected data from GUS), and sign consent forms. Three main issues arose regarding ethical approval and are summarized below.

#### Gaining Ethical Approval for Nonclinical Research

It was unclear which committee the SPACES project should be submitted to: the College of Social Science university ethics committee, as sponsors of the SPACES study, or the National Health Service (NHS) Research Ethics Committee (REC), as sponsors of the GUS longitudinal study. This lack of clarity led to the creation of applications to both committees and a lengthy period of correspondence between ScotCen, the University, and REC ethics advisors.

#### Informed Consent Procedures Were Markedly Different Depending on Committee

The two committees had different policies regarding Patient Information Sheets and Informed Consent Forms (ICF). This reflects the uncertainty and ambiguity that exists between taking part in medical research or traditional clinical trials and taking part in research more generally. Clinical Trial Regulations exist for the former, yet no real definitive process exists for the latter. The University of Glasgow committee required parental consent for all participating children younger than 18 years, in the view of the age of childhood according to common law as practiced in England, Wales, and Northern Ireland, whereas the NHS committee, operating within Scottish law, requested signed consent from children deemed competent to understand the process and from parents when deemed less competent. In England, no legal precedence or statute exists under common law for those younger than 16 years to give consent for medical treatment or research, although some treatment examples exist within case law (eg, Gillick case with respect to treatment, [15]). Scottish statute states that young people under the age of 16 years can give legally binding consent to participate in *medical* research as long as they are believed by the medical practitioner to be competent. The Guidance by the Medical Research Council [16], the funders of our study, states: “…It is not entirely clear whether this Scottish statute covers consent to participate in research…but in the absence of law dealing specifically with research, the principles of Scottish law relating to consent to procedures and treatment might be reasonably applied.”

#### Registration Packs Were Sent by Post to Participants and Included a Consent Form

The completion and return of this registration document (returning a paper copy by post, completion online, or completion in a phone call with study staff) allowed research equipment to be *sent* to participants. In most cases, a consent form was returned by post alongside the registration document—but not in all cases. To prevent doubling of resources and time, permission was granted by the ethics committee to send research equipment upon affirmation that the participant was *willing* to take part. However, research data could only be *included* in any analyses, where participants and parents both “opted in” to the study by returning a signed consent from. These were returned in a prepaid envelope.

**Figure 2:**
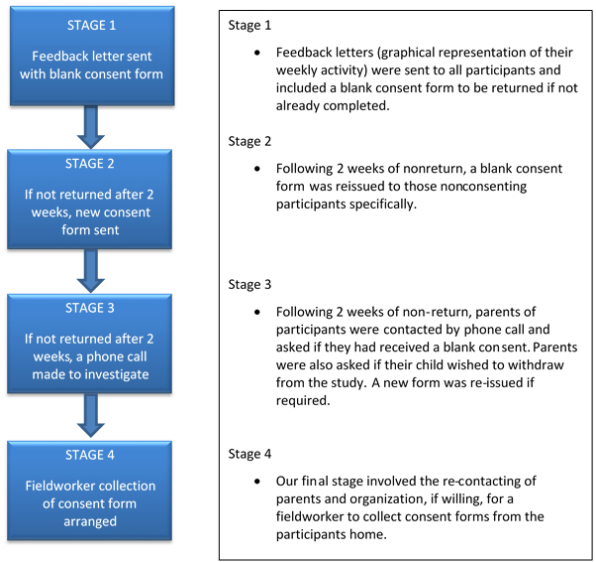
Staged process of retrieving missing consent forms.

Retrieval of missing consent forms was much more challenging than first anticipated. Our main concern was the increased likelihood of receiving valid data from participants without an accompanying consent form, thereby rendering the data unusable. Participants were given prepaid envelopes to return all study equipment, and in a number of cases (n=182), participants had forgotten to send a consent within this envelope. As such, the following process was created and implemented to retrieve those missing consent forms (see Figure 2). The costs associated with introducing a multistage process such as that in Figure 2 includes additional research assistant time costs to manage the preparation and sending of letters; the cost of the letters, postage, and packaging; and the field worker costs if physical collection is required. This final stage may not be possible in extremely large data collections that span across spatially diverse countries. For ethical reasons, the retrieval of consent forms is vitally important, and our study suggests that approximately 16.60% (182/1096) of participants were actively (by phone call or house visit) followed up.

### Recruitment

Participants had the choice to register for the study by returning the registration document by post, completing the registration online, or by making contact via a free phone number. Table 1 presents the recruitment sample by registration type. Approximately 20% (19.70%, 426/2162) registered by post, 4% (3.75%, 81/2162) online, and less than 1% (0.83%, 18/2162) actively called the study team to register.

As a result of poor return within the first wave of invitation letters, the study team made a decision within the first month of P1 that a further stage should be integrated, namely, a follow-up phone call if no registration document had been completed and returned within 2 weeks (see Figure 1). Approximately 2000 phone calls were attempted, with this stage resulting in the recruitment of a further 571 participants. Although a valuable opportunity to explain more about the study, this additional stage required extra resources, including field workers working out-of-office hours to contact the parents of participants.

### Study Equipment

**Figure 3:**
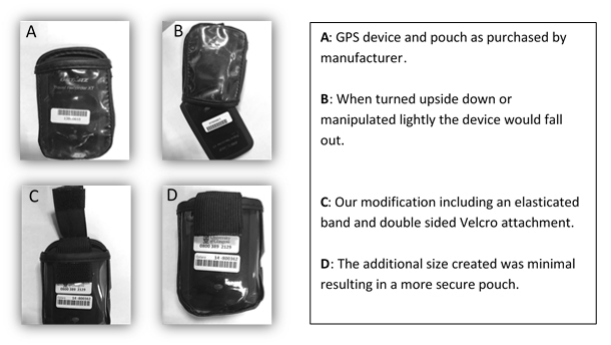
Demonstration of unsecure global positioning system (GPS) devices and our textile modifications to secure devices within their pouches. A: GPS device and pouch as purchased by manufacturer; B: When turned upside down or manipulated lightly the device would fall out; C: Our modification including an elasticated band and double sided Velcro attachment; and D: The additional size created was minimal resulting in a more secure pouch.

During our pilot work, it became evident that the pouches containing the GPS devices were of variable size, meaning that some GPS devices were not being securely held. Feedback from participants suggested that devices were falling out when the children ran or jumped. To resolve this issue, we fashioned an elasticated strap with Velcro attachments that ran across the opening of the pouch (Figure 3). This small modification prevented any further GPS devices from falling out of the pouch.

### Delivery of Study Materials

Reliance on a postal delivery method meant that we had to consciously consider the size of the items being delivered to ensure they would fit through a standard UK letterbox. During our pilot, we encountered an issue where the purchased GPS charging adapters were in fact too large (alongside the other pack contents) to fit through our pilot letterboxes, rendering these unusable for our project. No commercial plug by any major UK suppliers could be found to meet the project budget; however, we were able to source affordable smaller plugs from an Internet source in China. Upon a subsequent pilot phase—where study packs were sent to members of the study team to assess the postal delivery time and to ascertain whether they were successfully posted through the letterbox—the modified device packs were deemed suitable for the project.

**Table 1:** Response to take part in SPACES (Studying Physical Activity in Children’s Environments across Scotland) study.

Type of response	n (%), N=2162
Returned registration form in post	426 (19.70)
Online completion	81 (3.75)
Phone call from parent	18 (0.83)
Registered on phone	571 (26.41)
Withdrawal/refusal	337 (15.59)
Unable to contact	729 (33.72)

The delivery of devices was subject to two postal systems: the university internal system and the national Royal Mail system. SPHSU is an off-campus unit and is located at a distance of around 2 miles from the main university campus. As such, this had important practical and logistical considerations when sending out device packages to match participants’ preferred start dates: staff members from the main campus visited the off-site unit once each day to take all post back to be processed and sent. Subsequently, additional time (usually 1 day) had to be integrated into the physical process of delivering study materials as a result. As the project involved two postal systems, we increased the number of potential weak points in the chain, and this was particularly evident in relation to device loss. We seldom encountered issues when sending our device packages to participants, however, a number of participants were adamant that they had sent devices back to the unit, but these devices never arrived. From the 1096 device packages sent, we had 51 confirmed lost as missing 51 (4.65%), and a further 25 (total=76, 6.93%) presumed lost because of the inability to contact the participants in question or in cases where we were told the devices had been sent back but not arrived. This issue is not unique to the United Kingdom, and most research studies will consider using different methods of delivery for their study. An interesting comparison by Heath and Stewart [17], in a study of Australian football club members, found that an email approach was less expensive than a postal method (Aus $ 1.16 vs Aus $4.84 per useable response) and resulted in a faster response speed to the study (3.9 days vs 10.8 days). However, total response level for the postal method (46%) was more than double than that of the online method (21%) and was similar to that found in this study. Each research context will be different, and future work should consider all possible options and choose based on what most adequately suits their needs.

### Data Collection Phase

Our SMS message system seemed to work successfully, and feedback from a sample of parents was positive. The system was initially created to act as a reminder to wear the devices at the midpoint of the data collection; however, in a small number of cases the SMS acted as a prompt to the parent to inform us that the participant had yet to start the study period or had started a few days later than originally intended. Our system was built around the preferred start date of the participant, meaning that our SMS messages were either ineffective or had to be recalculated and resent. To enhance the effectiveness of this system, it may be beneficial to integrate a simple secondary process whereby parents can alert the research team when the data collection period has begun. This of course adds another stage of compliance and consideration should be given to the costs and benefits of doing so. Although we had strong compliance from the participants, future work may want to consider the benefits of increasing the SMS component to a daily reminder. This may be dependent on the population group or age, for instance if implementing a similar design with working adults or older populations. Doing so would involve a relatively small cost, so this should be considered and factored into a grant or funding proposal.

Our initial pilot work and subsequent protocol had organized for the GPS devices to record continuously at 10-second intervals for the 8 days of the study period (initially switched on by the participant). The default setting meant that the devices should have been able to record approximately 200,000 data points, however, with all of the aforementioned additional options selected (eg, visible satellites, elevation), we realized from the data recorded by the first few returned devices that the recordings were being stopped at day 7. In response, we sent updated instructions to all active participants to turn their devices off before going to bed and turn them back on upon waking. This enabled a full 8 days recording (memory capacity) but could have influenced the number of data points returned, as some participants may have forgotten to activate their devices on certain days.

### Data Processing Issues

We also encountered several software issues throughout the period of data collection and included a number of firmware updates (ActiGraph). Several updates created problems for the study. One particular update prevented access to the data stored on the device and was only solved by obtaining a customized piece of software from the manufacturer. This resulted in a delay of several weeks and hindered more collections and rewears during that period. A further firmware update resulted in reduced battery life for Bluetooth versions of the activity monitor. Fortunately, this issue was quickly spotted and it resulted in the loss of all ActiGraph data for only 5 participants.

A final unforeseen issue encountered in both activity monitors and GPS devices, arose as a result of UK daylight saving time (DST), where depending on time of the year, clocks either gain (October) or lose (March) an hour. Depending on when the devices were initiated and worn, the timestamps (ie, the date and hour/minute/second) of the downloaded data were incorrect by 1 hour. Where the GPS device software (QTravel) has an option to correct for DST when downloading and exporting the data, this option can be missed and is not necessarily well signposted. Even if this is selected, all timestamps are adjusted by 1 hour rather than only those recorded during DST. In addition, there appears to be a software bug in the correction across midnight that transitions back and forth across different times. This is an issue that will impact any research taking place in a geographical region that experiences DST. As such, this should be thought about in advance. Care should be taken when using this option and a few test cases should be pilot-tested in advance. A helpful paper by Hurvitz [18] is recommended reading for anyone using this type of data. A further issue discussed by Hurvitz relates to the issues that arise with the activity monitors during this period. The activity monitors are initiated by the software using the internal computer clock. As such, if these devices are initiated before the clocks changing, and the participants wear the device across the clock change, the file must be corrected to reflect this change. For the SPACES project, the activity monitor manufacturers were contacted, and they produced a piece of software that corrected all device files that were affected.

## Discussion

### Recommendations

Our experiences should be useful to any researcher who is planning to embark on a data collection study but may prove even more beneficial to those who implement similar designs. In general, the unanticipated challenges experienced as part of the implementation of the SPACES study resulted in substantial impact across four main categories: project timeline challenges; cost, budget and funding; participant engagement; and data challenges. Table 2 presents a summary of these issues in addition to suggested recommendations for researchers who may be planning similar projects. A major timeline issue that we experienced related to ethical approval. Our learning suggests that, within the UK context at least, only one ethics committee needs to review and approve an application. Provided they are a formally constituted body (whether through a Higher Education or a recognized Health Institute) then this will generally be sufficient for a research project. An important caveat is the distinction between clinical and nonclinical research projects involving the NHS. From a UK perspective, a nonclinical research project will usually be submitted to an ethics committee of the associated Higher Education institute. If the research is clinical in nature and is linked to the NHS (eg, potential participants are patients or users of the NHS, or the research would be require access to, or the use of, NHS premise), then formal applications should be submitted under the Department of Health/NHS framework.

Consent of underage children in all research is one that continues to be debated. With reference to this study, the issue was resolved by satisfying the College of Social Sciences ethics committee at the University of Glasgow, by having both parent and child complete and sign the ICFs. Ultimately, the process for our study ethics was ambiguous and complicated and very little legislation exists that deals specifically with children consenting to nonclinical trial-related research. If working with children younger than 18 years, and in partnership with an ongoing external study, it will be important to have this issue resolved at an early stage of the design development.

The cost implications for these types of studies require significant preplanning and forecasting, and all projects will have costed for the items such as personnel, instruments, and services. Our unit has a dedicated research support team with years of expertise in project management, budgeting, forecasting, and delivery, and this was a significant resource that made our data collection successful. We estimate that a project of this size and similarity would cost approximately £500k to conduct. Some considerations for future studies include the costs associated with database management of participants and study materials if necessary (ie, managing activity monitors and GPS devices); study documentation costs; data entry costs; and field work costs (eg, phone calls to participants). For those who use technology such as activity monitors or GPS devices, it is imperative that staff time is costed for device management (eg, charging, initializing, packaging, and downloading). Each device takes approximately 20 min to initialize and download. If you had 1000 participants in a study, this would “cost” approximately 330 hours. Future studies could use this information to cost their projects more realistically. One particular unanticipated example of a “cost” and “participant engagement” issue was that of project materials (GPS AC adapters) not passing through letterboxes. With this type of project design, we had to maximize the likelihood of participation, and this may have been reduced through the inconvenience of the respondents having to collect the package from a post office or collection office, especially for participants living in rural areas where this could be many miles away (in our study it might have meant a 40-mile round trip for some respondents).

**Table 2:** Issues experienced during study implementation, and suggested learning for future studies.

Project stage		Detail	Main implication	Suggested learning
**Recruitment (P1)**				
	Ethical approval	Advised to submit to more than one ethics committee	Timeline	It is not necessary to submit to more than one committee as long as the approving body is formally constituted. Consideration should still be given to which committee is chosen.
	Informed consent of minors	Informed consent process with children under 16/18 years of age is ambiguous and unclear	Timeline	Early discussion with an ethics committee is paramount. Guidance exists within the United Kingdom to assist with the decision-making process [16].
	Informed consent process	The postal delivery method created a logistical issue with gaining participant consent	Timeline	In discussion with an ethics committee, researchers may want to raise the option of two distinct consent processes: consent to take part, and consent for data to be used. This may allow the study to proceed while waiting for final signed consent. Consideration should also be given to developing a phone-based consent procedure (ie, recorded consent) if accepted by ethics committee.
	Participant recruitment	Success rates of registering varied by post, online, and by phone	Timeline; Budget	Active recruitment, where study staff can discuss, converse, and answer questions from participants, will result in greater numbers involved. Where possible, build the resources for this type of recruitment.
**Device pack (P2)**				
	Postal delivery method	Using the UK Royal Mail system and the University’s internal postal service extended the time needed to deliver and retrieve devices	Timeline	Researchers should consider how they will deliver the study materials to participants. If by post, multiple nodes and points of weakness may exist. These should be factored into the design.
	Study material delivery	If using a postal delivery method, study materials should be of size to fit through letterboxes	Participant engagement; Budget	Careful planning and pilot-testing of study materials should be conducted to ensure participants receive study equipment.
	Device loss	Physical equipment used in studies will be lost, broken, or go missing.	Budget, participant engagement	Minimize loss by ensuring all devices stay secure when being carried. Researchers should build in an anticipated loss of approximately 7% of devices if conducting a similar method.
**Technical issues**				
	Storage capacity	Study equipment (eg, global positioning system, GPS) should meet the storage requirements of the study period	Data; participant engagement	If using GPS devices, rigorous piloting that mimics full study conditions should be conducted to ensure all data can be recorded across study period.
	Software problems	Software glitches rendered some study devices inoperable	Data; Timeline	Consumer focused manufacturers should assist with faulty devices/software. It is also advisable to have access to software support within the study team.
	Daylight saving time (DST)	Devices that require an internal clock to regulate accurate recording will be impacted by DST	Data	Software/programming support will be beneficial to alter affected data. Alternatively, consideration should be given to stopping data collection around the changes in DST.

In addition, since we had purchased AC adapters in large quantities we added an unnecessary cost to the project by having to purchase more. Although specific to this study, we can extend our learning to any study that proposes to use a postal method to deliver study instruments or materials. We recommend that future studies—that plan on using similar delivery methods—consider and test whether their materials will be successfully delivered by fully pilot-testing these processes in the formative stages.

A final, “data” issue recommendation relates to the technical specifications of any device used in a study. Our decision was to collect location (GPS) data at a higher resolution, and as such we chose to keep the 10-second interval but to ask participants to turn their devices off during the night. Subsequently, we introduced a further step for the children involved in the study which could have impacted both participation in the study, as well as the quality of the data recorded. An alternative approach could have been to increase this to 15-second intervals, thus increasing storage capacity, or we could have decided against collecting so many of the additional options. Work by Schipperijn et al [12], for instance, has indicated that some of these additional variables may not be necessary to improve accuracy or assist with data cleaning and need not be recorded. However, we also suggest it is worthwhile to run analyses as part of the piloting process to inform the utility of these variables in specific study contexts.

### Conclusions

The purpose of this study was to describe the methods and unforeseen challenges experienced by the SPACES study, a nationally representative accelerometry and GPS data collection study in Scottish 10- to 11–year-olds children. The particular strength of this study is the description of our experiences with previously unidentified issues that can arise in studies of this design. Few reflective pieces exist within the literature, yet in preparing this study, we hope that this type of information will prove beneficial to those developing large-scale primary data collections, particularly those involving postal methods, as well as those using wearable technologies. We see a particular benefit during the funding proposal stage where information from this study can be used to build a more realistic picture of cost, particularly with regard to time and unforeseen staff costs. In conclusion, we hope that this study highlights some of the potential complications that can arise during studies of this nature with the desire of making other researchers aware, in advance, thereby allowing plans to be put in place to mitigate these issues.

## Appendix

Multimedia Appendix 1 SPACES information booklet and consent form given to participants.

Multimedia Appendix 2 SPACES registration document to take part in the study.

Multimedia Appendix 3 SPACES self-reported physical activity questionnaire (PAQ-C).

Multimedia Appendix 4 SPACES travel diary and device log book.

Multimedia Appendix 5 SPACES guidebook to manage the accelerometer and GPS device.

## Abbreviations

DST: daylight saving time
GPS: global positioning system
GUS: Growing Up in Scotland
ICF: Informed Consent Forms
NHS: National Health Service
PAQ-C: Physical Activity Questionnaire for Children
REC: Research Ethics Committee
SPACES: Studying Physical Activity in Children’s Environments across Scotland
SPHSU: Social and Public Health Sciences Unit
