# Supplementary material for: The Unanticipated Challenges Associated With Implementing an Observational Study Protocol in a Large-Scale Physical Activity and Global Positioning System Data Collection
Source: JMIR Res Protoc. 2018 Apr 30;7(4):e110. doi: 10.2196/resprot.9537 (PMC5952115; doi:10.2196/resprot.9537)
Supplement: Multimedia Appendix 1 [file resprot_v7i4e110_app1.pdf]

SPACES

Studying Physical Activity in Children's Environments across Scotland

Growing Up in Scotland

SPACES

PARTICIPANT INFORMATION BOOKLET

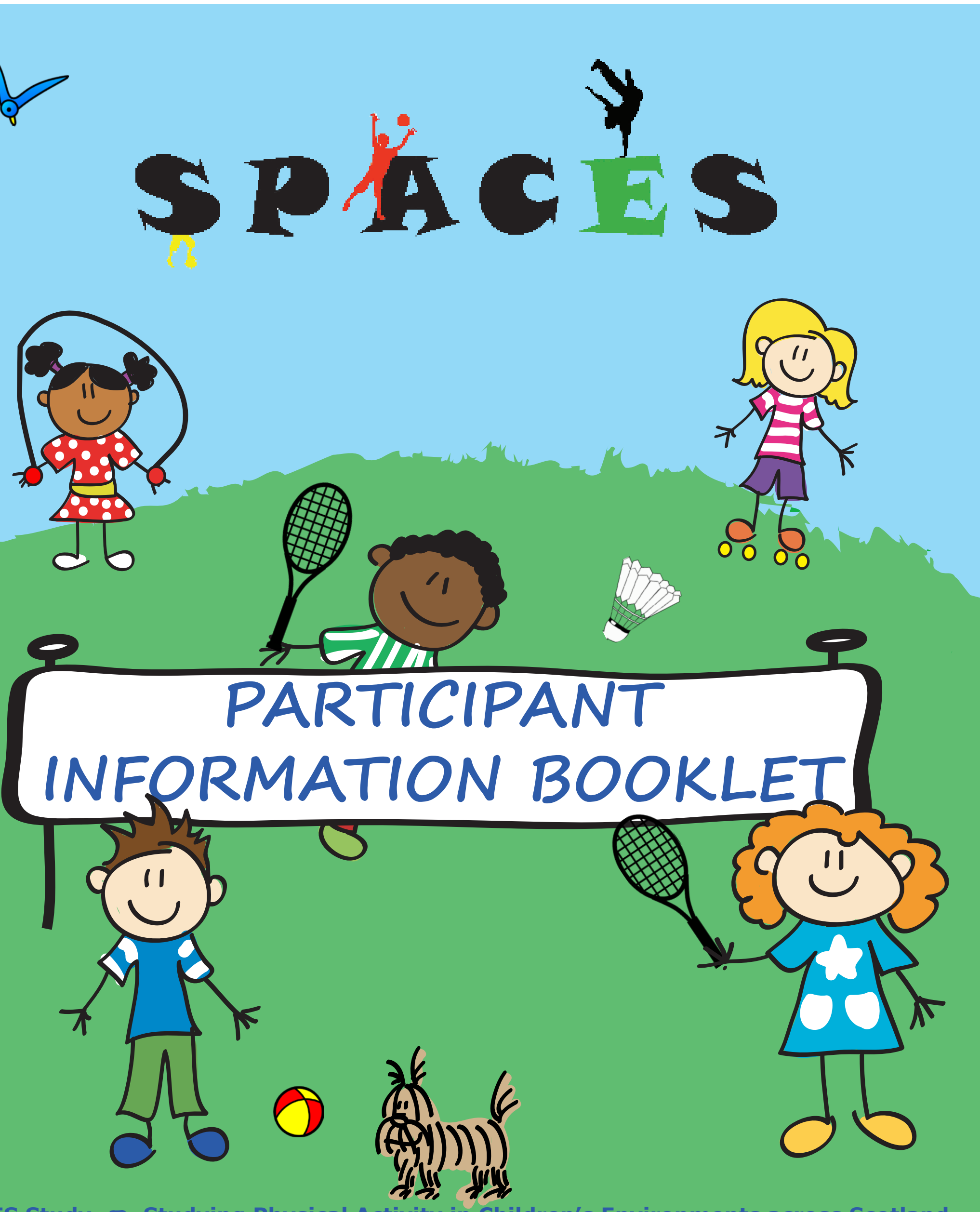

SPACES Study – Studying Physical Activity in Children's Environments across Scotland

What's SPACES all about?

You are being invited to take part in the SPACES study
(Studying Physical Activity in Children's Environments across Scotland)

The SPACES project is about helping us to understand more about young people's physical activity in their environment. To find this information out, we use pretty cool gadgets that tell us how active you are, and where that activity happens.

We are not directly part of the Growing up in Scotland (GUS) work you have been involved with, but we are working together with the GUS team.

This information booklet tells you about the important parts of the study. Please take the time to read the information carefully.

If it all seems like fun and you want to, then we would like you to take part. We can give you and your parent/carer a phone call to talk about taking part and this will allow you to ask any questions you may have. If you would like a phone call, please say so on the Registration Form we've sent you. Or you can do this online at www.sphsu.mrc.ac.uk/spaces-registration

There is a Consent form at the end of this booklet that you and your parent/carer will need to sign before we can use any data that you give us. Please fill this in and send it back to us in the enclosed FREEPOST envelope.

Who are the researchers?

Professor Anne Ellaway and Dr Paul McCrorie are the two main researchers involved in SPACES. We also have a team of other researchers and helpers, called the SPACES team, who will be involved with the project.

Why you?

We're trying to gather information about the amount of physical activity that boys and girls your age do. And because you are already taking part in the Growing Up in Scotland study, we thought you might be interested in taking part.

What do we need you to do?

What we'd like you to do is to wear two different gadgets for eight days. This will show how much activity you do, as well as where you do it. Both of these gadgets clip around your waist with an elastic belt.

The first device is called the ActiGraph activity monitor, and measures the energy that you use when you move about. It can also tell you how many steps you take. It is roughly the size of an ipod shuffle.

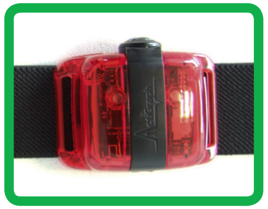

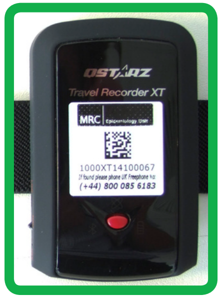

The second device is called the QStarz travel recorder and it measures where you are moving about.

These types of devices are usually called GPS devices. GPS is short for Global Positioning System. Both devices are attached to an elastic belt that is worn around your waist.

You wouldn't need to wear the belt all the time so we'd ask you to remove it just before going to sleep and put it on again once you wake up. You should not wear the belt and gadgets when you go swimming, wash (shower/bath) or take part in rough activities where it might get damaged (e.g. rugby or martial arts).

When you are wearing the belt, we would like you to note down any time that you take it off for longer than five minutes. The reason for this is to make sure that we don't think you were sitting down when it may just be that you were not wearing the belt.

We would also like you to note down when go to your bed and when you wake up. You will be given a little book to record this information, and it would be great if you could also tell us how you travel to and from school each day. There will be a special section for you to write these details down.

Don't worry about how to use the gadgets. If you want to take part there are instructions about how to use them. There will also be videos about how to use them if you visit our website: **spaces.sphsu.mrc.ac.uk**

We would like you to fill in a short questionnaire at the end of the study. It asks about the physical activities, sports and exercise you have been doing over the 8 days of the study. It will take about 20 minutes to finish. We want to make this easy for you to fill out, so you will have the choice of completing this on paper or online (we will give you a website address and login details to fill in the questionnaire online). This is something we will ask if you decide to take part.

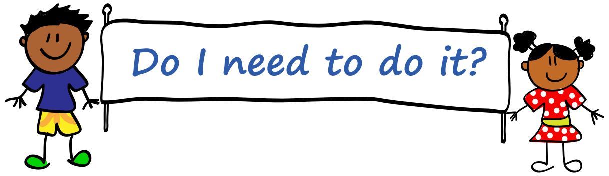

Do I need to do it?

No - it's totally up to you. If you don't want to take part then feel free to say no. If you decide to take part but then want to stop at anytime that's fine too.

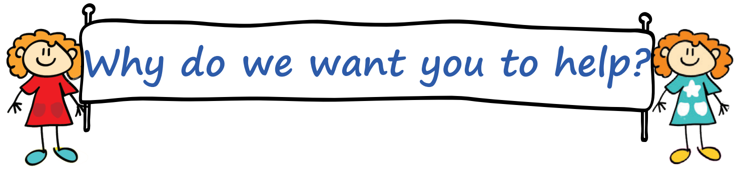

Why do we want you to help?

We want to see how much physical activity your age group does. For children your age, it's important to keep healthy as you grow older. Physical activity is a part of this process.

We also want to see what parts of the local area (like parks and streets near your home) are used by people of your age. By finding this out, we can make suggestions to the government and council that we hope will increase the number of people going outside.

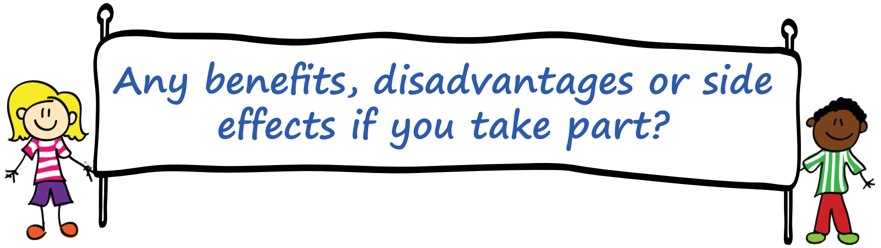

Any benefits, disadvantages or side effects if you take part?

You will be able to find out how much physical activity you do and where you tend to do it, which you may find interesting. After we have looked at the information on the devices we will send you some graphs showing you how active you were during the study.

I've heard about something called confidentiality. What's that?

Confidentiality is a word that is used to explain the privacy of your data, and is about who can see the information you give us and also how we will protect that information. There are things we are legally required to do that keep the data secret from anyone else who isn't allowed to see it.

For this study we will make sure of the following:

- Although we have a record of your name and address, we will store these details in a locked cupboard away from any data you give us during the study. Data is anything that you give us that can be seen as personal to you. For example, the physical activity or GPS information from your devices would be data.

> This is a complicated section so please ask your parent/ carer to explain if you are not sure

- As part of that process, any data you do give us will be given a unique number. This number will only be for you, and any information you give us will be given that number. There will be a 'key' that links that number to your name, but this will be locked away safely and only special members of the SPACES team will be able to see this. These people will not be able to see your data.

- If you decide that you don't want to take part at any time then that's not a problem. If you would like us to delete any data collected up to that point then please let us know.

Other data collected by Growing Up in Scotland (GUS)

You will have provided lots of data over the years to the GUS project. As part of the current study, we would like to ask if it would be OK to use some of this data in our study. This would include things such as your most recent height and weight measurements, household income, how many people stay in your house, and how many brothers or sisters you have. We may use other information collected by GUS as well.

To be clear, the information we will use can only be seen by people on the SPACES team - nobody else will be allowed to see this.

What happens at the end of the study?

At the end of the study we plan to do a few things both with the data you give us, and the results of the study.

- As you have been kind enough to help with our study, we would love to give you some feedback. This will include how much activity you carried out, and also some information from the overall study itself.

> This is another complicated section so please ask your parent/ carer to explain if you are not sure

- We plan to write reports, using the results of the study, so that other people can find out about the research. Again, it is important to understand that anyone who reads these reports will not know who you are.

It's important that you understand that the GPS data you give us will never be presented or used for results where you can be identified personally. It is also important for you to know that we do not monitor where you go at the same time as you are there. The data we see is in the past.

The data that you give us will be useful to other people, and because of that we want to ask your permission for it to be used by other **real** researchers that we trust. When we use the term 'data' in this instance, we mean your physical activity data and GPS data, as well as a map location of your home, and school. This would also include other data such as if you are a boy or a girl.

- The physical activity data will be stored with the UK Data Service, in a way that stops you from being identified. The UK Data Service has an excellent reputation and your data will be stored securely and confidentially.
  http://ukdataservice.ac.uk

- The GPS and physical activity data will also be stored for about 50 years at our unit, the Social and Public Health Sciences Unit (SPHSU), University of Glasgow, which is the workplace of both Professor Anne Ellaway and Dr Paul McCrorie. SPHSU already hold large data-sets on their secure computer servers, and the rules of the unit adhere to the Data Protection Act 1998, which includes strict levels of security and privacy for your data.

- The only way anyone can access the data is if they can be trusted, and that they agree to protect your identity and data. By signing the consent form, you are giving us permission to make that decision. Your name, date of birth or home address would not be shared with anyone else.

Who is organising and funding the research?

The SPACES project is funded by the Chief Scientist's Office (CSO), the Medical Research Council (MRC) and the Scottish Government. The study is being carried out by scientists from the MRC's Social and Public Health Sciences Unit (SPHSU) in Glasgow, who specialise in public health research. We are based at the University of Glasgow.

Also, members of the GUS team will be helping us out at some stages. They have given you some information about the study already.

Has anyone looked at the study?

As researchers, we need to make sure our research is carried out correctly. The people who make sure it is correct are called ethics committees. The research we want to carry out has been looked at by an ethics committee at the University of Glasgow, and the National Research Ethics Service (NRES), the team who look at the work carried out by GUS.

How to contact us

If you have any questions or queries about taking part you can contact us on FREEPHONE 0800 389 2129, at our offices on 0141 353 7500, or by email at **spaces@sphsu.mrc.ac.uk** and we will make sure that one of the research team gets in touch with you as soon as possible. You can also find more information about SPACES at **spaces.sphsu.mrc.ac.uk**

Can I speak to someone if I have any concerns?

If you want to speak to someone who is aware of the study, but not directly involved in the research team, or if you have any concerns or complaint, you can contact Dr Muir Houston, the University of Glasgow's College of Social Sciences Ethics Officer at 0141-330-4699, or email **socsci-ethics@glasgow.ac.uk**

Consent Form

Title of project: Studying Physical Activity in Children's Environments across Scotland (SPACES) Study

Name of researchers: Professor Anne Ellaway and Dr Paul McCrorie

Please initial boxes below

	Child	Parent Carer
We confirm that I have read and understood the Participant Information Booklet for the above study.		
We have had the opportunity to ask questions and for those questions to be answered satisfactorily.		
We understand that participation is voluntary and that the participant is free to withdraw at any time, without giving any reason.		
We understand that the data collected from the Actigraph and GPS device will be stored securely with all personal details removed, and agree for it to be held as set out in the Participant Information Booklet.		
We agree that the data collected within this study may be shared with other genuine researchers, as set out in the Participant Information Booklet.		
We agree to the SPACES project using previous data collected as part of Growing Up in Scotland, and understand that this data will not be shared with anyone else.		
I agree to take part in the above study.<br>I consent to my child taking part in the above study.		

Participant **NAME** ____________________

Participant **SIGNATURE** ____________________

Parent/Guardian **NAME** ____________________

Parent/Guardian **SIGNATURE** ____________________

Date ____________________

After both you and your parent/carer have filled in and signed the consent form, please **carefully**

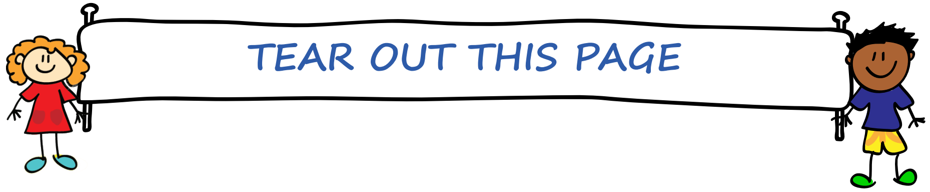

and send it back to us in the enclosed FREEPOST envelope.

Then, please **fill in** and **sign** the copy of the consent form which comes next. It will stay in this booklet for you to keep, so that you and your parent/carer will know what you signed.

Consent Form (copy)

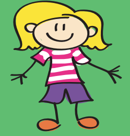

Title of project: Studying Physical Activity in Children's Environments across Scotland (SPACES) Study

Name of researchers: Professor Anne Ellaway and Dr Paul McCrorie

Please initial boxes below

	Child	Parent Carer
We confirm that we have read and understood the Participant Information Booklet for the above study.		
We have had the opportunity to ask questions and for those questions to be answered satisfactorily.		
We understand that participation is voluntary and that the participant is free to withdraw at any time, without giving any reason.		
We understand that the data collected from the Actigraph and GPS device will be stored securely with all personal details removed, and agree for it to be held as set out in the Participant Information Booklet.		
We agree that the data collected within this study may be shared with other genuine researchers, as set out in the Participant Information Booklet.		
We agree to the SPACES project using previous data collected as part of Growing Up in Scotland, and understand that this data will not be shared with anyone else.		
I agree to take part in the above study.<br>I consent to my child taking part in the above study.		

Participant **NAME** ____________________

Participant **SIGNATURE** ____________________

Parent/Guardian **NAME** ____________________

Parent/Guardian **SIGNATURE** ____________________

Date ____________________

WORDSEARCH

B	P	K	A	Y	P	G	L	U	H	R	X	Y	S	O	N	C
L	T	I	U	A	W	T	N	E	T	B	A	L	L	F	B	K
C	Y	T	E	N	N	I	S	S	N	H	Q	X	Z	V	N	X
F	R	R	P	J	N	C	Z	W	H	D	X	N	H	N	J	V
H	Y	G	O	L	L	A	B	T	O	O	F	Q	H	T	W	B
I	I	N	O	U	K	B	K	K	O	J	S	R	H	I	J	M
Z	N	I	B	H	N	G	O	L	F	A	M	O	V	B	G	L
K	O	N	I	Y	D	D	C	L	E	Z	Y	R	G	T	L	A
C	T	N	A	Z	W	H	E	C	V	L	S	N	K	A	E	C
K	N	U	Q	X	O	G	R	R	N	E	I	N	B	M	W	Z
X	I	R	A	T	K	I	Y	I	S	M	H	T	O	D	R	F
W	M	D	N	W	C	M	B	P	M	L	E	L	Y	Z	L	Q
K	D	V	J	K	K	K	K	I	C	K	Y	B	N	Y	T	Y
W	A	P	E	H	Y	M	W	G	S	Z	M	M	U	A	F	C
W	B	T	N	U	R	S	C	A	F	H	I	H	O	E	L	J
T	D	Y	H	D	P	J	B	Z	Y	E	G	U	A	T	M	Q
N	D	M	I	Z	V	G	M	R	J	F	F	B	S	S	E	J

badminton

basketball

golf

netball

swimming

tennis

cricket

football

rounders

running

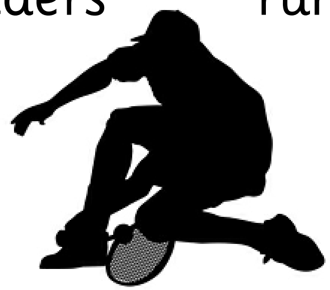

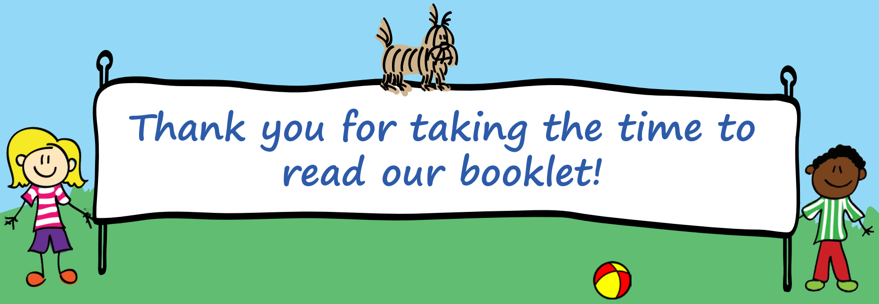

MRC/CSO Social and Public Health Sciences Unit
University of Glasgow
200 Renfield street
Glasgow G2 3AX
Tel: 0141 353 7500 www.sphsu.mrc.ac.uk

A Research Unit supported by the Medical Research Council
and the Chief Scientist Office of the Scottish Government
Health Directorates, at the University of Glasgow

The University of Glasgow, charity number SC004401
