# Supplementary material for: The Unanticipated Challenges Associated With Implementing an Observational Study Protocol in a Large-Scale Physical Activity and Global Positioning System Data Collection
Source: JMIR Res Protoc. 2018 Apr 30;7(4):e110. doi: 10.2196/resprot.9537 (PMC5952115; doi:10.2196/resprot.9537)
Supplement: Multimedia Appendix 2 [file resprot_v7i4e110_app2.pdf]

«IDNO»

Use this for online registration login

Growing Up in Scotland

SPACES

Studying Physical Activity in Children's Environments across Scotland

«LCname»
«Address1»
«Address2»
«Address3»
«Address4»
«Postcode»

March 2015

Dear «LCname»

Studying Physical Activity in Children's Environment across Scotland (SPACES)

We are writing to let you know about an important new study which we hope your child (**«Cname»**) will take part in. The central focus of the research is to investigate how active Scottish children are. We also want to investigate how factors such as access to green space and recreational facilities influence the physical activity levels of boys and girls.

The project is funded by the Scottish Government, the Medical Research Council, and the Chief Scientists Office. The study will be supported by Scotcen and the Growing Up in Scotland study, and has been approved by Glasgow University, and the Scottish Government.

The SPACES study involves wearing an activity monitor and a Global Positioning Systems (GPS) device over **eight** consecutive days. The activity monitor is small and unobtrusive and is worn via an elastic band around the waist and measures the energy used when moving around; the GPS device attaches itself to the same waist band and can measure the physical location of a person at set times.

As part of the project, participants will be asked to fill out a physical activity questionnaire that records the types of activities engaged in during the previous seven days. In addition, we would like to link the data collected in the SPACES study with that which has already been collected by GUS.

We do hope you will be able to support this important research. There is a detailed information booklet within this pack that you and your

MRC/CSO Social and Public Health Sciences Unit, University of Glasgow,
200 Renfield Street, Glasgow, G2 3AX
Tel: 0141 353 7500 www.gla.ac.uk/sphsu

son/daughter should read. As part of the process, we can phone you to provide more information, and allow you the opportunity to ask questions about the study. We understand that this may not be needed once you have read the information booklet, so please indicate on the attached registration document if you would like us to phone and explain the study further.

We need to receive the completed registration document before we can send out the GPS and activity monitor, for which a FREEPOST envelope is enclosed. Alternatively, you can complete this form online at **https://www.sphsu.mrc.ac.uk/spaces-registration** if more convenient. Within the information booklet there is also a consent form that we would like you and your child to complete and send back within the FREEPOST envelope.

If you want to get in touch with the team, please call us on **0800 389 2129** (9am – 5.30pm). If you are put through to our answer machine, please leave your name, number and best contact time, and we will get back to you as soon as possible.

If you have any concerns or other comments about the study, please contact Dr Muir Houston, the Ethics Officer for the University of Glasgow's College of Social Sciences at **socsci-ethics@glasgow.ac.uk**

Yours sincerely,

Professor Anne Ellaway
Head of Neighbourhoods and Health Programme

MRC/CSO Social and Public Health Sciences Unit, University of Glasgow,
200 Renfield Street, Glasgow, G2 3AX
Tel: 0141 353 7500 www.gla.ac.uk/sphsu

Registration Document

PLEASE COMPLETE THE NEXT SECTION IF YOU **DO NOT** REQUIRE A PHONE CALL FROM ONE OF THE SPACES STAFF MEMBERS

Before we can send the devices out for your child to wear, we

I agree to the activity monitor and GPS device being sent to

To ensure we send out an appropriate sized belt for

The size of belt required for my son/daughter is (pl

up to 65cm 65 – 75cm 75 – 85 cm

In our experience, there is a point during our studies where participants begin to forget t

I agree to being sent a text/email midway through the study to remind my son/daught

Mobile number ______________________________

Email address ______________________________

We aim to send out all packages on a Monday or Tuesday of each week. This usually means that the devices should arrive on a Wedn

The best date for my son/daughter to start wearing the devices would be (i.e. Friday, March 3rd)

Please remember that you can phone the SPACES freephone number on **0800 389 2129** (9 am – 5.30pm) if you have any questions or queries.
