# Supplementary material for: The Unanticipated Challenges Associated With Implementing an Observational Study Protocol in a Large-Scale Physical Activity and Global Positioning System Data Collection
Source: JMIR Res Protoc. 2018 Apr 30;7(4):e110. doi: 10.2196/resprot.9537 (PMC5952115; doi:10.2196/resprot.9537)
Supplement: Multimedia Appendix 3 [file resprot_v7i4e110_app3.pdf]

Studying Physical Activity in Children's Environments across Scotland

Growing Up in Scotland

SPACES

We are trying to find out about your level of physical activity from **the last 7 days** (in the last week). This includes sports or dance that make you sweat or make your legs feel tired, or games that make you breathe hard, like tag, skipping, running, climbing, and others.

Remember

1 There are no right or wrong answers
— this is not a test.

2 Please answer all the questions as honestly and accurately as you can
— this is very important.

University of Glasgow

The University of Glasgow, charity number SC004401

MRC Medical Research Council

Chief Scientist Office

1 **Physical activity in your spare time:**

Have you done any of the following activities in the past 7 days (last week) and if so, how many times?

Please tick **ONE BOX on each line**

	No	1 – 2 times	3 – 4 times	5 – 6 times	7 times or more
Skipping	☐	☐	☐	☐	☐
Rowing/canoeing	☐	☐	☐	☐	☐
Roller skating/blading	☐	☐	☐	☐	☐
Tag/Chases	☐	☐	☐	☐	☐
Walking for exercise	☐	☐	☐	☐	☐
Bicycling	☐	☐	☐	☐	☐
Jogging or running	☐	☐	☐	☐	☐
Aerobics	☐	☐	☐	☐	☐
Swimming	☐	☐	☐	☐	☐
Baseball, softball	☐	☐	☐	☐	☐
Dance	☐	☐	☐	☐	☐
Rugby	☐	☐	☐	☐	☐
Badminton	☐	☐	☐	☐	☐
Skateboarding	☐	☐	☐	☐	☐
Football	☐	☐	☐	☐	☐
Tennis	☐	☐	☐	☐	☐
Volleyball	☐	☐	☐	☐	☐

Please tick **ONE BOX on each line**

	No	1 – 2 times	3 – 4 times	5 – 6 times	7 times or more
Hockey	☐	☐	☐	☐	☐
Basketball	☐	☐	☐	☐	☐
Ice skating	☐	☐	☐	☐	☐
Skiing/Snowboarding	☐	☐	☐	☐	☐
Ice hockey	☐	☐	☐	☐	☐
Other (please **write in**)					
______	☐	☐	☐	☐	☐
______	☐	☐	☐	☐	☐

2 **In the last 7 days, during your physical education (PE) classes, how often were you very active (playing hard, running, jumping, throwing)?**

Please tick **ONE BOX only**

- I don’t do PE ☐
- Hardly ever ☐
- Sometimes ☐
- Quite often ☐
- Always ☐

3 **In the last 7 days, what did you do most of the time AT INTERVAL/BREAK?**

Please tick **ONE BOX only**

- Sat down (talking, reading, doing schoolwork) ☐
- Stood around or walked around ☐
- Ran or played a little bit ☐
- Ran around and played quite a bit ☐
- Ran and played hard most of the time ☐

4 **In the last 7 days, what did you do most of the time AT LUNCH (besides eating lunch)?**

Please tick **ONE BOX only**

- Sat down (talking, reading, doing schoolwork) ☐
- Stood around or walked around ☐
- Ran or played a little bit ☐
- Ran around and played quite a bit ☐
- Ran and played hard most of the time ☐

5 **In the last 7 days, on how many days RIGHT AFTER SCHOOL did you do sports, dance or play games in which you were very active?**

Please tick **ONE BOX only**

- None ☐
- 1 time last week ☐
- 2 or 3 times last week ☐
- 4 times last week ☐
- 5 times last week ☐

6 **In the last 7 days, on how many EVENINGS did you do sports, dance or play games in which you were very active?**

Please tick **ONE BOX only**

- None ☐
- 1 time last week ☐
- 2 or 3 times last week ☐
- 4 times last week ☐
- 5 times last week ☐

7 **ON THE LAST WEEKEND, how many times did you do sports, dance, or play games in which you were very active?**

Please tick **ONE BOX only**

- None ☐
- 1 time ☐
- 2 – 3 times ☐
- 4 – 5 times ☐
- 6 or more times ☐

8 **Which ONE of the following describes you best for the last 7 days? Read ALL FIVE statements before deciding on the ONE answer that describes you.**

Please tick **ONE BOX only**

A All or most of my free time was spent doing things that involve little physical effort. ☐

B I sometimes (1 – 2 times last week) did physical things in my free time (like played sports, went running, swimming, bike riding, did aerobics) ☐

C I often (3 – 4 times last week) did physical things in my free time ☐

D I quite often (5 – 6 times last week) did physical things in my free time ☐

E I very often (7 or more times last week) did physical things in my free time ☐

9 **Mark how often you did physical activity (like playing sports, games, doing or any other physical activity) for each day last week.**

Please tick **ONE BOX on each line**

	None	Little bit	Medium	Often	Very often
Monday	☐	☐	☐	☐	☐
Tuesday	☐	☐	☐	☐	☐
Wednesday	☐	☐	☐	☐	☐
Thursday	☐	☐	☐	☐	☐
Friday	☐	☐	☐	☐	☐
Saturday	☐	☐	☐	☐	☐
Sunday	☐	☐	☐	☐	☐

10 **Were you sick last week, or did anything prevent you from doing your normal physical activities?**

Please tick **ONE BOX**

Yes ☐

No ☐

If YES, what prevented you?

Please **WRITE IN**

____

THANK YOU

Please ensure you have tried your best to complete all 10 questions and that you have not missed any by mistake.
