# Supplementary material for: The Unanticipated Challenges Associated With Implementing an Observational Study Protocol in a Large-Scale Physical Activity and Global Positioning System Data Collection
Source: JMIR Res Protoc. 2018 Apr 30;7(4):e110. doi: 10.2196/resprot.9537 (PMC5952115; doi:10.2196/resprot.9537)
Supplement: Multimedia Appendix 4 [file resprot_v7i4e110_app4.pdf]

SPACES

Studying Physical Activity in Children's Environments across Scotland

Growing Up in Scotland

SPACES

Waist Belt Log

and

Travel Diary

ID NO.

How long should I wear my devices?

We would like you to wear the WAIST belt for 8 days, and for as close to 12 hours per day as you can manage.
We understand that you may not always be able to wear the belt for 12 hours a day (like at the weekend), but if you wear it for much less than 12 hours on any day we may have to ask you to wear it again.

When you send your devices back we will download a graph which will show us how long you wore them each day.
Below are two example graphs. The one on the left is from a person who wore the waist belt for a long enough time.
The one on the right is from a person who didn't wear the belt for long enough and we had to ask them to wear it again.

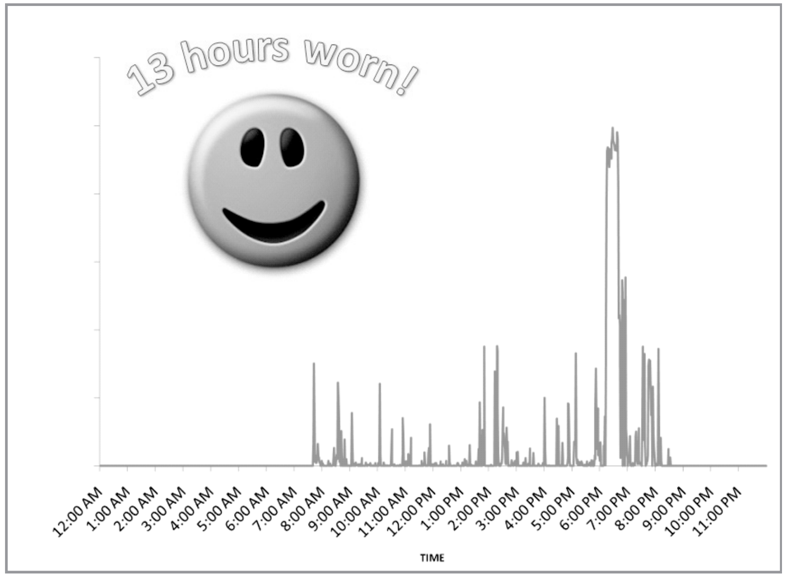

This person did a great job of wearing their waist belt! As you can see on the graph, they put the device belt on when they woke up at about 7:30 in the morning, and kept it on until about 8:30 at night.

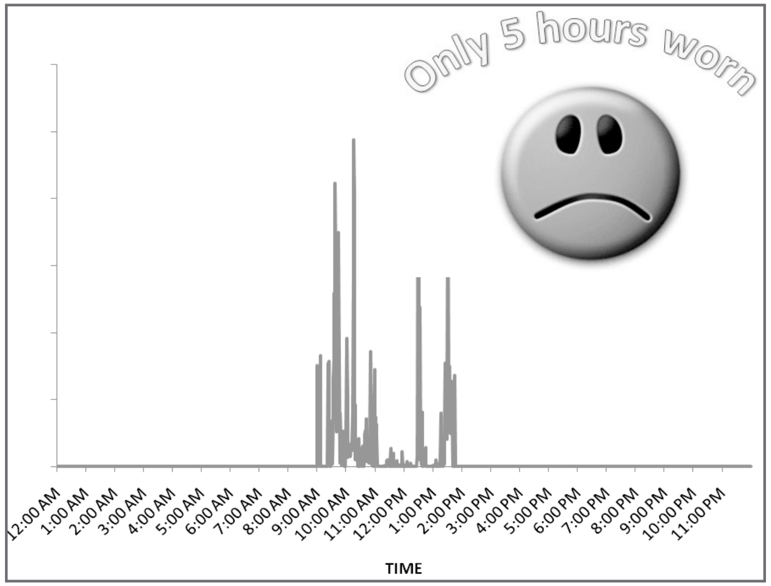

This person would have to wear the devices again. They put the waist belt on when they woke up at about 9:00 in the morning, but took it off at about 2:00pm

Waist Belt Log

It is important that you wear the waist belt for 8 consecutive days.

Please do this for each day of the study.

On the next two pages, write down the times when you put the belt on in the morning and take it off at night.

We also would like to know if you take the belt off for longer than 5 minutes at any point during the day. Please note the time and reason why you took the belt off.

The example on the right illustrates the process.

Example

**Day** 1	Thursday
Date	05/03/15
Time belt was put on in the morning	07:30
Time belt was taken off at night	10:45

During the day

Time off	12:45
Time on	13:30
Reason	Swimming
Time off	:
Time on	:
Reason	
Time off	:
Time on	:
Reason	

Day 1

Date

Time belt was put on in the morning :

Time belt was taken off at night :

During the day

Time off :

Time on :

Reason

Time off :

Time on :

Reason

Time off :

Time on :

Reason

Day 2

Date

Time belt was put on in the morning :

Time belt was taken off at night :

During the day

Time off :

Time on :

Reason

Time off :

Time on :

Reason

Time off :

Time on :

Reason

Day 3

Date

Time belt was put on in the morning :

Time belt was taken off at night :

During the day

Time off :

Time on :

Reason

Time off :

Time on :

Reason

Time off :

Time on :

Reason

Day 4

Date

Time belt was put on in the morning :

Time belt was taken off at night :

During the day

Time off :

Time on :

Reason

Time off :

Time on :

Reason

Time off :

Time on :

Reason

Day 5

Date

Time belt was put on in the morning :

Time belt was taken off at night :

During the day

Time off :

Time on :

Reason

Time off :

Time on :

Reason

Time off :

Time on :

Reason

Day 6

Date

Time belt was put on in the morning :

Time belt was taken off at night :

During the day

Time off :

Time on :

Reason

Time off :

Time on :

Reason

Time off :

Time on :

Reason

Day 7

Date

Time belt was put on in the morning :

Time belt was taken off at night :

During the day

Time off :

Time on :

Reason

Time off :

Time on :

Reason

Time off :

Time on :

Reason

Day 8

Date

Time belt was put on in the morning :

Time belt was taken off at night :

During the day

Time off :

Time on :

Reason

Time off :

Time on :

Reason

Time off :

Time on :

Reason

Travel Diary

We want to understand more about how you travel to and from school each day during the week. To do so we would like you to fill in a diary. Here is how we would like you to do it:

1. For each day of the week think about how you travel to school, and also how you travel back from school.
2. Count the total amount of time you spend travelling and break that down into the specific parts of the journey.
   - **a** By ‘parts’ we mean things like walking, travelling by bus, and by car.
   - **b** There are 9 choices to choose from - you don’t need to use all of them.
3. Write the amount of time you spend in each part under the pictures of that way of travelling .

   For example, if it takes you 30 minutes to get to school, and 10 minutes of that is walking, write 10 under minutes in the ‘walking’ minutes section. If the remaining 20 minutes is spent on a bus then write 20 under minutes in the bus section. Don’t worry if you come home by a different method. In the example below, the person travelled all the way home by car and it took 20 minutes, and you write this in the ‘From school’ row.
4. Make sure you put in the times as accurately as you can for each individual part of your journey.
5. Don’t forget! Make sure to fill this in for **each day** of the week AND for the journey **TO** school, and **FROM** school.

Remember to fill in **to** and **from** each day

Example

Minutes and hours go in separate boxes

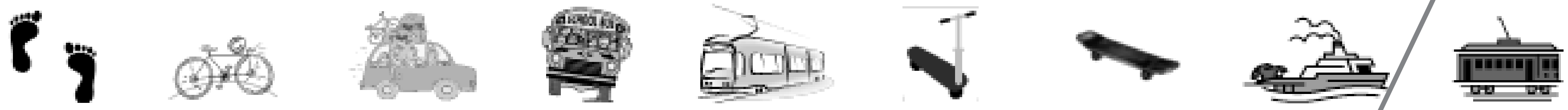

		Walking		Cycling		Car		Bus		Train		Scooter		Skateboard		Ferry		Tram	
		Hours	Minutes	Hours	Minutes	Hours	Minutes	Hours	Minutes	Hours	Minutes	Hours	Minutes	Hours	Minutes	Hours	Minutes	Hours	Minutes
MONDAY	To school		10						20										
	From school						20												

Remember
Two journeys per day!

Day	Journey	Walking Hours	Walking Minutes	Cycling Hours	Cycling Minutes	Car Hours	Car Minutes	Bus Hours	Bus Minutes	Train Hours	Train Minutes	Scooter Hours	Scooter Minutes	Skateboard Hours	Skateboard Minutes	Ferry Hours	Ferry Minutes	Tram Hours	Tram Minutes
MONDAY	To school																		
	From school																		
TUESDAY	To school																		
	From school																		
WEDNESDAY	To school																		
	From school																		
THURSDAY	To school																		
	From school																		
FRIDAY	To school																		
	From school																		

Remember
Two journeys
per day!

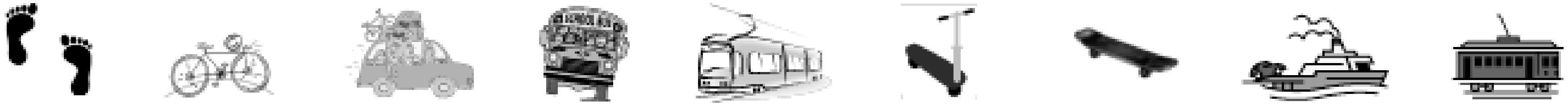

		Walking		Cycling		Car		Bus		Train		Scooter		Skateboard		Ferry		Tram	
		Hours	Minutes	Hours	Minutes	Hours	Minutes	Hours	Minutes	Hours	Minutes	Hours	Minutes	Hours	Minutes	Hours	Minutes	Hours	Minutes
MONDAY	To school																		
	From school																		
TUESDAY	To school																		
	From school																		
WEDNESDAY	To school																		
	From school																		
THURSDAY	To school																		
	From school																		
FRIDAY	To school																		
	From school																		
