# Supplementary material for: The Unanticipated Challenges Associated With Implementing an Observational Study Protocol in a Large-Scale Physical Activity and Global Positioning System Data Collection
Source: JMIR Res Protoc. 2018 Apr 30;7(4):e110. doi: 10.2196/resprot.9537 (PMC5952115; doi:10.2196/resprot.9537)
Supplement: Multimedia Appendix 5 [file resprot_v7i4e110_app5.pdf]

SPACES

Studying Physical Activity in Children's Environments across Scotland

Growing Up in Scotland

HANDBOOK

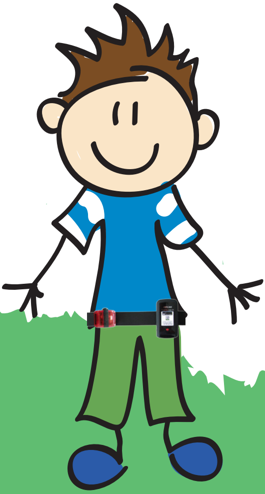

Contact us

ADMIN

Elaine Hindle
Elaine.Hindle@glasgow.ac.uk
0800 389 2129

Website spaces.sphsu.mrc.ac.uk

Freephone 0800 389 2129

RESEARCH

Professor Anne Ellaway
Anne.Ellaway@glasgow.ac.uk
0141 353 7500

Dr Paul McCrorie
Paul.Mccrorie@glasgow.ac.uk
0141 353 7500

For this study we will ask you to wear two devices attached to a belt. At first, the devices may feel slightly awkward, but most people soon forget about them. It's extremely important that you wear the devices as instructed.

- The smaller device on the belt measures the movement of the hips
- The larger device on the belt measures your location, speed and distance you travel
- Neither device records what you say so please don't worry about that
- Please wear the waist belt for 8 consecutive days

A Guide to the Lights on the GPS Device

LED Symbol	Light status	Description and colour of light
Battery	Flashing	Low Battery
	Solid	Recharging
	Off	Fully charged
Bluetooth	Flashing	Shouldn't occur
	Solid	Shouldn't occur
	Off	Should be in this state
GPS	Flashing	Recording satellite position normally
	Solid	Trying to find satellites, not yet found
	Off	Device is switched off
Log	Flashing	• Every 2 seconds - Low memory (20%)<br>• 3 times in a row - Point of Interest button has been pressed
	Solid	Memory is full
	Off	Switch on the side is not positioned at 'LOG'

FAQ QStarz Travel Recorder

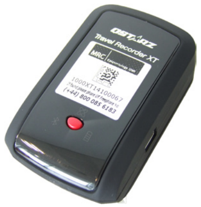

What is the QStarz Travel Recorder?

The QStarz Travel Recorder is a small device that uses Global Positioning Systems (GPS) technology to record where you are active. As you move about outside, the device records those positions every couple of seconds.

What is GPS?

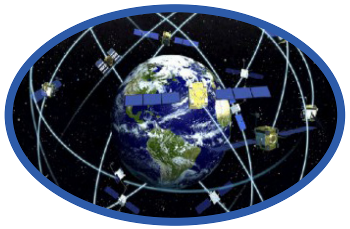

GPS is a cool technology that uses a number of objects in space. These are called satellites and they are able to talk to your device. There are 24 satellites that travel around the Earth during the day and the QStarz device is able to speak to them if it can see them in the sky. The more of these that the device can see, the better it is at recording where you are. It's the same technology that's used in smart phones for the maps app.

Where should the device be positioned?

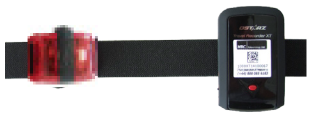

The device is worn alongside the ActiGraph activity monitor. It should be worn on the **opposite** side to the Actigraph - meaning it should be worn over your **left** hip bone.
As you will be wearing it alongside the ActiGraph, we would like you to wear it for **eight** consecutive days.

Do I need to charge the device?

Yes. You will be given a charging cable that should be used with the device. We need you to charge the device every night. A useful habit to get into is to attach the charging cable when you remove the belt before bed.

ActiGraph

FAQ

What is the ActiGraph?

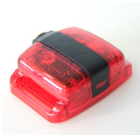

The **ActiGraph** is a small device that measures how much you move. In particular, it is good at identifying how much energy you use in day to day activities (e.g. walking, playing sport, dancing).

Where should it be positioned?

The best position for the device is above the right hip bone. Clip the belt around your waist and then slide the device around until it is just above your right hip bone and pointing upwards as in this picture.

The device will not work properly in pockets or bags.

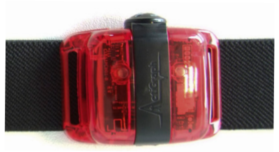

Can the device be worn under clothing?

The device can be worn under, or on top of clothing - whatever is most comfortable. The most important thing is to make sure that the device is positioned fairly tightly against the body so as to prevent it from moving about.

How long has the device to be worn?

To make sure we can gather enough information, we would like you to wear the monitor for **eight** days in a row, and it would be great if you could keep it on for at least 12 hours each day. If you can't manage this we might need to ask you to wear the device again for the missing time.

Should the device be worn all the time?

It should only be worn when you are awake. This means that you should take the belt off when you go to bed and put it back on when you wake up.

The device is not waterproof. It should be OK if some water splashes on it but please take it off when going for a bath/ shower or swimming. It should also be removed when you take part in activities where it might get hit hard, such as rugby or martial arts. Other sports like football, hockey and basketball should be fine.

What happens if the device is lost, stolen or broken?

If the device is lost, stolen or broken we would appreciate if you could contact us as soon as possible. The devices themselves are expensive and we have other projects that need them after this one. If you believe that you have lost the device we would ask for you to have a good look for it in all the places it may have been.

Can the battery run out?

No. The battery is inside the device and will last for more than eight days so there's no need to worry about the battery running out.

Is anything supposed to flash on the device?

No.There shouldn't be any lights flashing on the device when it is recording. If it flashes red or green at all, please contact us as soon as possible.

Wearing the devices

Device Belt – proper placement and use

The belt should be worn snugly around your waist, either over or under your clothing (whichever is more comfortable).

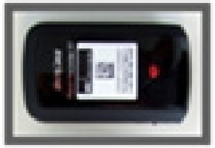

Position the larger black device on the belt above your LEFT hip bone.

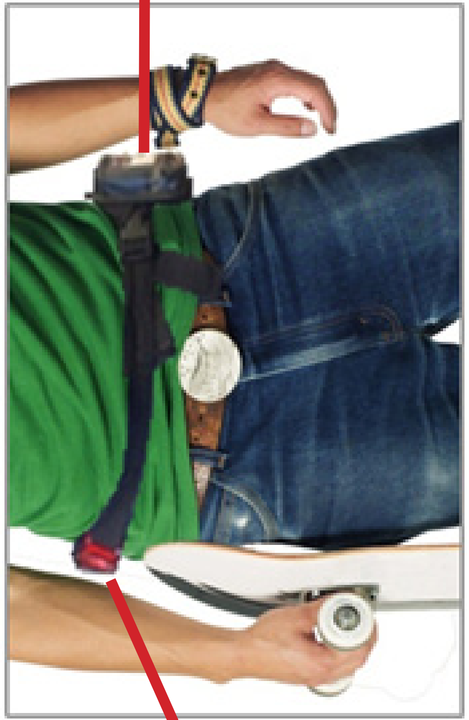

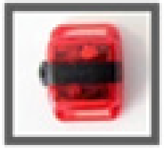

Position the small red device just above your RIGHT hip bone.

You can check the correct placement of the belt devices by letting your arms hang down by your sides. Each device should be in line with your arms, just touching your forearms.

Tips for wearing the belt

Put the waist belt on first thing in the morning

Try not to get it wet
(take if off when swimming & bathing)

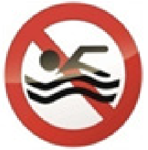

There are no 'ON' or 'OFF' switches on the activity monitor

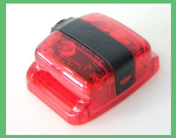

Take the waist belt off just before you go to bed and charge the LARGE BLACK device

Do not let anyone else wear the belt

Try to wear the waist belt for 12 hours every day for all 8 days. This isn't always possible but please try.

The GPS device will arrive switched off. You must push the switch to 'LOG' mode before starting the study.

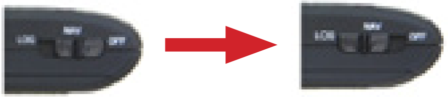

Charging the GPS device

Charge every night.

Plug the charger into a wall socket and attach the device with the USB cable.

Remember to put the belt on every morning & complete your waist belt log.

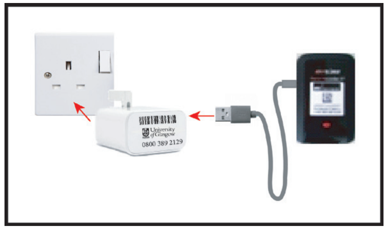

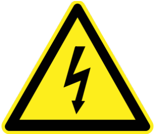

- Don't let leads from plugged-in devices trail across the floor
- Never stick anything into the holes of an electric socket – you could get a shock.

CHECKLIST

When to do what!

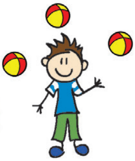

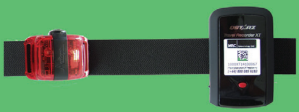

BELT WITH TWO DEVICES ATTACHED

Wear it as soon as you can after it arrives in the post.

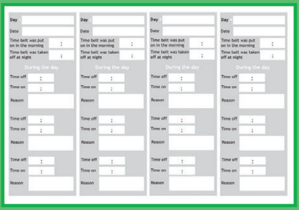

WAIST BELT LOG

Fill in the times that you put the belt on and take it off. Try to do this at the start and end of each day.

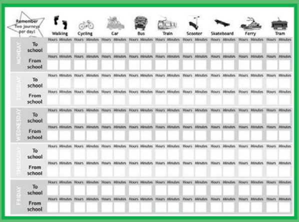

TRAVEL DIARY

Fill in how you got to and from school at the end of each day.

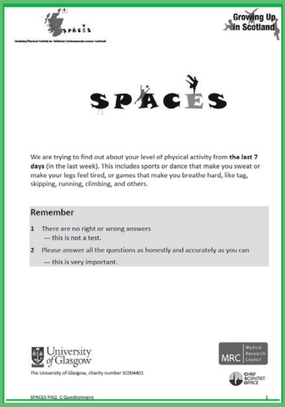
Growing Up in Scotland

SPACES

We are trying to find out about your level of physical activity from the last 7 days (in the last week). This includes sports or dance that make you sweat or make your legs feel tired, or games that make you breathe hard, like tag, skipping, running, climbing, and others.

Remember

1 There are no right or wrong answers — this is not a test.

2 Please answer all the questions as honestly and accurately as you can — this is very important.

University of Glasgow

MRC Medical Research Council

CHIEF SCIENTIST OFFICE

SPACES PAQ_C Questionnaire 1

PAQ-C QUESTIONNAIRE

After you have worn the belt for 8 full days, fill in the physical activity questionnaire.

Fill in the paper version which came with this pack
OR
Fill in the online version

www.sphsu.mrc.ac.uk/spaces-questionnaire

To fill in online you will need your postcode and your ID number (on the front of this handbook).
